# Three-Dimensional Printed Knee Implants: Insights into Surgeons’ Points of View

**DOI:** 10.3390/jpm13050811

**Published:** 2023-05-10

**Authors:** Mathieu Le Stum, Thomas Bertin, Myriam Le Goff-Pronost, Claire Apremont, Guillaume Dardenne, Ghislaine Rolland-Lozachmeur, Eric Stindel

**Affiliations:** 1 Laboratoire de Traitement de l’Information Médicale (LATIM), UMR 1101, Faculté de Médecine de Brest, Université de Brest, Université de Bretagne Occidentale, 29200 Brest, France; 2 Laboratoire de Traitement de l’Information Médicale (LATIM), UMR 1101, Institut National de la Santé et de la Recherche Médicale, INSERM, 29200 Brest, France; 3 Laboratoire de Traitement de l’Information Médicale (LATIM), UMR 1101, M@rsouin, Institut Mines-Telecom, IMT Atlantique, 29200 Brest, France; 4 Laboratoire de Traitement de l’Information Médicale (LATIM), UMR 1101, Centre Hospitalo-Universitaire de Brest, CHU Brest, 29200 Brest, France

**Keywords:** 3D-printed implant, discourse analysis, knee, orthopedic, surgeon’s point of view

## Abstract

Three-dimensional printing is a technology that has been developed and applied in several medical specialties, especially orthopedic surgery. Knee arthroplasty is the most commonly performed procedure. To fit the morphology of each knee, surgeons can choose between different standardized off-the-shelf implant sizes or opt for customized 3D-printed implants. However, routine adoption of the latter has been slow and faces several barriers. Existing studies focus on technical improvements or case studies and do not directly address the surgeon’s perspective. Our study invited surgeons to express themselves freely and answer the question “What do you think about the manufacture of a prosthesis by 3D printing?”. The questionnaire was completed by 90 surgeons. On average, they had more than 10 years of experience (52, 57.8% ± 10.2%), worked in public hospitals (54, 60% ± 10.1%), and performed between 0 and 100 prostheses per year (60, 66.7% ± 9.7%). They also reported not using planning software (47, 52.2% ± 9.7%), navigation systems, or robots (62, 68.9% ± 9.6%). Regarding the use of technological innovation, they agreed on the extra surgical time needed (67, 74.4% ± 9.0%). The answers obtained were classified according to two criteria: (i) opinions, and (ii) motivations. Among the respondents, 51 (70% ± 9.5%) had positive and 22 (30% ± 9.5%) had negative opinions about 3D printing. The motivations were distributed among seven categories (surgery, materials, costs, logistics, time, customization, and regulatory) and mainly related to “pre-surgery” and “post-surgery” concerns. Finally, the results showed that the use of navigation systems or robots may be associated with a more positive view of 3DP. The purpose of our study was to examine knee surgeons’ perceptions of 3DP at a time of significant expansion of this technology. Our study showed that there was no opposition to its implementation, although some surgeons indicated that they were waiting for validated results. They also questioned the entire supply chain, including hospitals, insurance companies, and manufacturers. Although there was no opposition to its implementation, 3D printing is at a crucial point in its development and its full adoption will require advances in all areas of joint replacement.

## 1. Introduction

Three-dimensional printing (3DP), also known as additive manufacturing, is a technology first developed by Charles W. Hull in the early 1980s. Although it has been known for over 40 years, its development has advanced rapidly in the last decade and it is now considered to be part of the third technological and industrial revolution [1,2]. Based on different technologies (stereolithography, selective laser sintering, etc.) and materials (plaster, metal, plastic, ceramics, etc.), 3DP technology has been applied in various industries such as aerospace, education, automotive, architecture, pharmaceutical, and medical [1,2,3,4].

In medicine, the use of 3DP utilities has developed and grown exponentially in recent years in several specialties, including dentistry, maxillofacial surgery, neurosurgery, orthopedics, and traumatology. This technology allows the production of models or replicas, customized tools or surgical guides (Personalized Surgery Instruments (PSI), etc.), customized implants, drugs, and biocompatible tissues [2,5]. This is the result of improved machine accessibility and easy-to-use software [6]. Among the medical fields using 3DP, joint surgery is the most studied (45.18% of the studies), followed by maxillofacial (24.12%), cranial (12.72%), and spinal (7.46%) surgeries. Around two-thirds of 3DP are used for PSI and customized implants (CIs) account for around 10% [7].

In the medical field, orthopedic and traumatology surgery was one of the earliest adopters of 3DP, as it is one of the most dynamic specialties, with rapid and innovative advances in treatment and surgery [6,8]. In this field, 3DP has seen great success due to the ease of medical image processing, as it mainly involves bone structures that provide clear visibility and contrast in computed tomography [9]. Thanks to the use of 3D planning software, the images are processed, analyzed, and then translated into a virtual 3D model, which replicates all patient’s anatomy to allow the virtual design of a replica, PSI, or customized implant (CI). Once validated by surgeons, this model is sent for 3DP (layer by layer) and finishing. In terms of customized implants, this technology is designed to reproduce the native anatomy of the joint, unlike standard methods that use a series of manufactured standard sizes [8]. This printing technology has several advantages, such as replicating the patient’s own geometry, saving time, and decreasing production costs [1,2]. The disadvantages of this technology are related to the mechanical properties of the printed implant, the loss of information on soft tissue (cartilage, ligament, etc.) that can influence bone cuts, and the prolonged postprocessing process [1,6,8].

In knee surgery, total knee arthroplasty is the most commonly performed procedure. However, the results are unsatisfactory in 20% of cases and between 8 and 12% of patients ask for revisions [10,11,12]. The main reasons for revisions are infection, mechanical loosening (including instability and malalignment), and polyethylene wear [11,12,13]. Theoretically, these problems could be minimized by a more individualized strategy since the positioning and fit of knee prostheses determine the functional outcome for patients. Nowadays, to fit the morphology of each knee, surgeons mainly use conventional, off-the-shelf implants, which come in different sizes and models. These implants are based on standardized anthropometric measurements, which cannot take into account all individual characteristics [14,15]. To address this issue, CIs were developed using 3D-printing technology. Several studies have shown the potential benefits of CIs in improving mechanical alignment, implant fit, bone coverage (overhang/underhang) and restoration, bone preservation, knee strength, range of motion, and axial rotation [16]. However, some recent studies of knee CIs have produced conflicting results [14,17]. Despite their potential advantages, their adoption in the operating room has been slow, as they face several barriers related to (1) surgeon practices such as initial training, familiarity, and comfort with off-the-shelf implants, and (2) costs and insurance coverage [16]. To the best of our knowledge, custom knee implants are still in development and are mainly used for patients who present less conventional anthropometric characteristics [7,14,16].

In the majority of studies, mostly focusing on PSI, medical outcomes were reported to be improved with the use of 3DP [1]. However, the existing studies on CIs focused only on the technical improvements or costs. Additionally, they analyzed isolated clinical cases or case studies and did not include comparative analyses with traditional implants [7,13,15,17]. Moreover, a publication bias may explain these results, with positive results being more commonly published than negative ones [1]. Furthermore, issues related to implementation by surgeons were rarely considered.

Given the potential advantages of 3DP over conventional processing technologies, its limited adoption by surgeons is thus questionable. To the best of our knowledge, no study has addressed the surgeon’s point of view. However, just as patient preferences are yet to be taken into consideration in evidence-based medicine [18,19], a better understanding of the discrepancy between observed technological developments and their slow adoption in practice seems of paramount importance. This is why this study aimed to identify surgeons’ thoughts regarding 3DP. This approach is in line with that of numerous other works (in social sciences) that investigate, question, and analyze users’ opinions and their social representations about various phenomena that influence habits, including the emergence of a new technology.

## 2. Material and Method

### 2.1. Questionnaire

A questionnaire was randomly addressed to French surgeons (1) via an online survey and distributed through the French National Orthopedic Society (Sofcot), and (2) at a regional congress (SOO-Western Orthopedic Society) between January and June 2022. The questions were developed based on a literature review and insights were gained from interviews with representative experts (four orthopedic surgeons, a cognitive scientist specializing in technology adoption, and a rehabilitation physician). It was divided into five sections: (I) sociodemographic information, (II) current surgical practices, (III) preferences, (IV) thoughts on using an innovative 3D prosthesis, and (V) affinity for interacting with technology. To achieve our objective, we focused on the first two sections and the unique open-ended question in Section III, which allowed surgeons to freely express their opinions. Indeed, they had to answer the question, “What do you think about manufacturing a prosthesis using 3DP?”. The ethics committee was not required since no personal data were collected.

### 2.2. Categorization of Answers

To this common approach used in public health, a complementary approach rooted in language science was introduced.

We performed a discourse analysis [20] of the entire corpus of answers to the open-ended question from Section III. This analysis took into account the lexicon (which words were used?), semantics (to refer to what?), syntactic parameters (sentence configuration),d enunciative point of view (how does the surgeon express his views?). The original French text of the answers given as examples below and its translation into English can be found in Appendix A.

This led us to establish a main criterion for categorizing surgeons’ opinions (strongly positive, strongly negative, weakly positive, and weakly negative) and a secondary criterion for justifying each opinion (whether it was supported by a motivation or not). For instance, while one respondent answered, “Not interested, this is not the right target” (strongly negative/motivation), others responded with “Brilliant” (strongly positive/no motivation) or “Don’t see the point” (strongly negative/no motivation).

Surgeons who indicated they were strongly positive either explicitly agreed with the 3DP solution or provided a positive motivation in support of 3DP (sometimes both). Those who indicated they were strongly negative either explicitly disagreed with the 3DP solution or provided a negative motivation against 3DP (sometimes both). Those who indicated they were weakly positive either formulated a weak agreement or expressed a positive opinion that was immediately challenged by a contrary motivation usually introduced using “but” or “if” (French words: “mais” and “si”). Those who indicated they were weakly negative either formulated a weak disagreement or expressed a skeptical opinion supported by negative motivations.

In the second step, the motivations were categorized into seven categories as follows. Surgery included motivations related to the surgical procedure itself and its planning. The other six motivations were lexically expressed in a very transparent way: costs, logistics, materials, time, regulatory, and customization (Table 1).

Finally, we classified the motivations into surgical phases: pre-operative, intra-operative, and post-operative. Pre-operative included motivations related to the preparation of the fitting of the prosthesis before the surgical act. Intra-operative included motivations related to the procedure itself and post-operative included motivations related to the strength of the material and the follow-up (Table 2).

### 2.3. Statistical Analysis

Descriptive statistics were used to describe data with a 95% confidence interval and a 5% alpha risk. The categorical variables extracted from the questionnaire were related to sociodemographic information and current surgical practices.

For statistical analysis, both strongly and weakly positive opinions were grouped as positive, and both strongly and weakly negative opinions were grouped as negative. The Chi-squared test of independence was used on the descriptive statistics to test whether the main criterion (i.e., positive or negative) was related to the categorical variables.

Logistic regression was used to describe the influence of the categorical variables on the primary outcome of interest. The variables were adjusted for each other. ANOVA was used to assess the significant effect of the variables on the model.

Statistical tests were performed using the R version 4.0.2 software and corresponding packages (questionR, gtsummary).

## 3. Results

### 3.1. Demographics

A total of 90 surgeons answered the questionnaire. All were male and most had more than 10 years of experience (52, 57.8% ± 10.2%), worked in public hospitals (54, 60% ± 10.1%), and performed between 0 and 100 prostheses fittings per year (60, 66.7% ± 9.7%). They also mostly reported not using planning software (47, 52.2% ± 9.7%), navigation systems, or robots (62, 68.9% ± 9.6%). Regarding the use of technological innovation, most surgeons agreed to additional surgical time (67, 74.4% ± 9.0%). As part of their patient follow-up, most surgeons also reported a desire for sensors to be integrated into the implant (66, 73.3% ± 9.1%).

### 3.2. Opinions Expressed

Out of the 90 questionnaires received, the open question was left unanswered 14 times. In addition, three responses explicitly stated that they had no opinion (e.g., “aucun avis”, *no opinion*). Although these responses may be viewed as ironic (for instance, a surgeon wrote “RIEN” in capitals, which is French for “NOTHING”), we decided to discard them to avoid the risk of misinterpretation.

The remaining 73 responses were categorized according to our main criterion: 51 (70% ± 9.5%) were positive (43.8% ± 10.3% strongly positive and 26% ± 9.1% weakly positive) and 22 (30% ± 9.5%) were negative (15.1% ± 7.4% strongly negative and 15.1% ± 7.4% weakly negative). No relationship was found between the categorical variables and the opinions (positive or negative) (*p* > 0.05) according to the experience, working structure, number of prostheses per year, and use of planning software. The *p*-value was significant for the variables, “use of navigation or robotics” and extra surgical time (Table 3).

**Figure 1:**
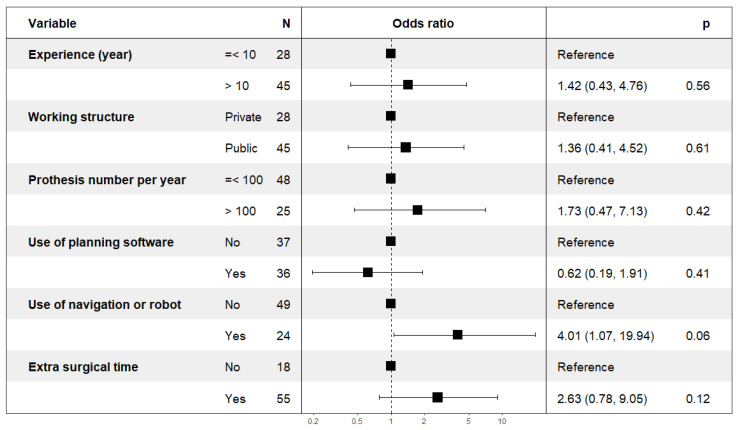
Results of the logistic regression to measure the probability of the positive opinions relative to the negative opinions. The exact *p*-value for “use of navigation or robot” is *p* = 0.056.

Logistic regression showed that the “use of navigation or robot” was associated with more positive views on 3DP, with an odds ratio *p*-value result of 0.056 (Figure 1, rounded to 0.06), which needs some discussion. There was no impact of the variable “extra surgical time” (*p* ≥ 0.1). A significant effect of the variable “use of navigation or robot” on the model (*p* = 0.039), unlike the variable “extra surgical time” (*p* ≥ 0.1), was demonstrated by the complementary ANOVA analysis. This implies a confounding relationship between these two variables related to “extra surgical time”.

An explicit motivation (secondary criterion) was expressed in 39 (53.4% ± 11.4%) answers, whereas the remaining 34 (46.6% ± 11.4%) answers did not provide any motivations. Of the responses providing motivations, 61.5% (24) were accompanied by positive opinions and 38.5% (15) were accompanied by negative ones (Table 4).

### 3.3. Expressed Motivations Related to the Justification of Opinions

When focusing on the various categories of motivation, surgery was the most expressed motivation (39.1% of the responses), followed by materials (15.2%), costs (10.9%), logistics (10.9%), time (8.7%), customization (8.7%), and regulatory (6.5%). The positive motivations focused more on surgery and customization, whereas the negative motivations focused more on surgery and materials (Table 4).

Pre-operative (58.5% of the responses) and post-operative (31.7%) were the most common motivations when focusing on the stage of surgery. The intra-operative stage was barely mentioned (9.7%) (Table 4).

Beyond statistics, the syntactic study showed that most of the strongly positive responses were rather brief and expressed no motivations at all (e.g., “excellent; it may be the future”). A few of them supported their enthusiasm with motivations (e.g., “good idea given the speed of the prosthesis manufacturing process”). A unique response provided a raw positive motivation instead of an opinion (e.g., “Interest in customization”).

Out of the rarer strongly negative answers, most of them dismissed 3DP technology rather harshly (e.g., “useless; insignificant”). The remaining responses supported the negative opinions with motivations (e.g., “don’t think it’s useful (time-consuming + costly)”) or provided solely negative motivations (e.g., “what is the patient’s normal knee”).

Most of the weakly positive answers combined two elements: (a) spontaneous confidence (b) counterbalanced by concerns (e.g., (a) “is a good solution” (b) “if it meets current standards”). Such answers show that many surgeons are open to the 3DP perspective and are willing to be convinced of its benefits. A few weakly positive answers formulated a raw prudent opinion (e.g., “not bad”).

Finally, weakly negative answers did not dismiss 3DP explicitly but expressed skepticism. They consisted of (i) a focal point to be addressed (e.g., “fragility; lifespan issue”), (ii) a question to be answered (e.g., mechanical strength?), (iii) a doubt to be dispelled (e.g., “dubious about the quality of materials”), or (iv) a direct expression of circumspection (e.g., “prudence, prudence”).

## 4. Discussion

In our study, two-thirds of surgeons had “positive opinions”, whereas one-third had “negative opinions”. Predominantly, they had more than 10 years of experience, worked in the public sector, and performed a maximum of 100 prostheses per year. Regarding their use of technology, both groups typically did not use navigation or robotic systems but were prepared to add extra time to their surgeries. However, there was an equal number of users and non-users of planning software (Table 3).

Statistical analysis showed that experience, working structure, number of prostheses per year, use of planning software, and extra surgical time did not influence the opinions. However, as a variable, the use of navigation or robotic systems may be questionable. With a significant overall effect, its exact influence needs to be clarified. We can assume a significant relationship between its use and a positive opinion of 3DP (*p* = 0.056) (Table 3 and Figure 1). A larger sample size is necessary to determine its exact influence.

For two decades, the use of assisted technologies (Computer-Assisted Orthopedic Surgery (CAOS), which includes navigation systems or robots) to reduce complications and help surgeons in knee arthroplasty has increased (+154% from 2008 to 2015). Compared to other joints, knee arthroplasty is the main application, with 18% of knee surgeons using it in 2015 [21,22]. This proportion is expected to reach 32% by 2032 [23]. More specifically, the use of robots by hospitals has increased by over 500% from 2009 to 2013 [24]. Compared to PSI and conventional methods, CAOS and robots have demonstrated better accuracy and precision as far as component positioning is concerned. Nevertheless, the functional results of CAOS and robots in comparison to conventional methods are still controversial [22]. As a result of these assisted technologies, extra surgical time has been noticeable, averaging 15–25 additional minutes for TKA [24]. Surgeons who are already using these technological tools are, therefore, accustomed to the additional time required for the surgical procedure. This would explain the observed confounding effect between the additional time devoted to the placement and the use of technology such as CAOS (e.g., navigation) or robotic systems.

Due to the unique morphology of each patient, it is crucial to have good positioning of the CI. Correct placement will result in good functional outcomes that meet the anatomical needs of patients. However, difficulties have been reported in the accurate placement of CIs. Surgeons can use tools such as navigation systems or robotics to replicate the same resection plane used in the design software to achieve the correct fit [25]. However, the majority of studies that address the use of navigation or robotic systems have been conducted in an academic setting or with previously trained surgeons. For these individuals, expertise and familiarity with these technologies may be a factor in the achievement of favorable outcomes. In the hands of a less experienced surgeon, these technologies may provide unproven benefits [26]. Designing a CI involves many steps, including using numerous imaging and prosthesis design software programs, which are not always easy to use [25]. In our pilot study, this experience-based point of view was supported by the emergence of two potential user groups based on their use or non-use of navigation or robotic technologies.

The ways they expressed their opinions of 3DP were the secondary results. It was either expressed directly (i.e., without any associated explicit motivation) or associated with an argument and thus expressed a motivation. “positive opinions” were mainly concerned with the anatomical and technical aspects of the surgical procedure, and, to a lesser extent, with the financial, material strength, or time-saving aspects. “negative opinions” were more likely to be influenced by normality (i.e., standardization) surrounding the surgical procedure, material strength, and, to a lesser extent, the regulatory issues, administrative delays of order, or financial issues. The study also showed that surgeons primarily focused on the pre-surgical phase (with mainly surgical procedure concerns) and the post-surgical phase (with mainly “material” strength and lifespan concerns). These observations indicate that physicians focus more on the environment of the surgical act. The procedure itself is rarely an issue.

In our study, we observed that an undecided (i.e., weakly convinced) population exists, which requires more in-depth results. Therefore, 3DP is an expected technology within the surgeon community.

To better understand the “weakly convinced” concerns regarding the use of 3DP (and thus CIs), it would be appropriate to look at the barriers mentioned. The technological nature of their words suggests that for surgeons, the surgical outcomes are the most important of these barriers. However, to obtain a complete picture, other concerns, such as cost, administrative barriers (public procurement, regulatory approval, etc.), and training, should be addressed. Our study did not allow us to weigh these motivations against each other.

Our results are consistent with those of previous studies showing that CI adoption depends on several factors. The most important of these are surgeons’ recommendations [16]. Because there is little evidence to show that CI improves patient outcomes compared to OTS implants, surgeons remain in a “wait and see” position and continue to use their current practices. Further clinical trials that demonstrate the long-term superiority of CI are thus expected [14,17,27,28]. Additionally, improvements would be more robust by integrating sensors into the implants, as requested by surgeons, and taking into account patient preferences [19]. Furthermore, there will be a time lag before we observe the better performance and use of new products [16]. A balance must also be ensured between surgeons’ workloads (before and during surgery) and the safety aspect of the implants (wear and tear) [29]. These positions are highlighted by the “weakly convinced” responses in our study.

In addition to this main factor, which is directly related to surgeons’ expertise, several other external factors have to be taken into consideration. Financial considerations, such as total costs, contracts between hospitals and insurance companies, or OTS implant manufacturers, may influence the acceptance of CIs [16]. Short-term goals, such as ensuring known surgical outcomes with OTS implants for insurance or hospitals and market share for vendors, have to be balanced with long-term goals [16]. A cost-effectiveness study would be useful to compare 3DP and OTS implants [29]. In fact, although technological developments (e.g., printers, software, and materials) have allowed a reduction in the cost of producing CIs (mainly through outsourcing to manufacturers), there has been an increase in costs linked to the additional processing time before surgery (e.g., conversion of 2D images into 3D, individual analysis of the joint, planning of the operation). Compared to OTS, 3DP-designed and manufactured implants require more interactions between surgeons and engineers [1,6,8]. The expected future growth of revision surgery resulting from the actual burden of primary joint arthroplasty is the perfect illustration of a long-term goal [30]. In this case, the adoption of CIs and the expected outcomes could lead to improvements in patient outcomes (intra- and post-operatively) and, consequently, a reduction in revision surgery [27,28]. A higher adoption rate could lead to a decrease in costs for hospitals and an increase in market share for manufacturers [16,31]. Our study uncovered these concerns, although they were not predominant.

Another barrier is related to the surgery itself, with the need to maintain backup implants in case of problems with CIs or a potential increase in malpractice liability (with legal risks or administrative issues). This leads us to the last obstacle, which is the surgeons’ overall preferences for OTS implants due to their training, familiarity, and comfort with them [16]. Since it is accepted that 3DP in orthopedics has a promising future, surgeons are encouraged to consider its use. However, this requires adequate pre-operative planning and the use of software, as well as other advanced skills in understanding 3DP [32]. The need for education in this rapidly changing field is therefore growing. In addition to training, facilitating its use, particularly through the use of artificial intelligence or predictive models, could be another way to address these adoption challenges [8]. The latter could, for example, make it easier to preprocess images and reduce the time needed to process them. Such technologies are currently under development.

Indeed, surgeons do not have to worry if the clinical benefits of 3DP are not realized as intended. Innovation comes with the adoption and subsequent critical evaluation of technology [26].

The main limitation of our study was the administration of the questionnaire to academic or research-oriented knee surgeons, which may have introduced a selection bias. As this was in the context of a pilot study, we focused on knee surgeons to address the research project to which this study is attached (the FollowKnee project). The aim of this project is the development of a new chain of care for customized and connected knee prostheses. In light of this pilot analysis, a comparison with the opinions of surgeons specializing in other joints could be of interest. In this way, the opinions expressed about 3DP could be differentiated according to the respective joint. Distributing this pilot questionnaire directly to a larger panel of surgeons in hospitals could also reduce the selection bias.

However, we can also consider the respondents as opinion leaders, as they are mostly already convinced and can contribute significantly to the diffusion of this technology. Another limitation was the lack of contextualization of the responses due to the lack of an interview component. This may have been helpful for better classifying the opinions and motivations. Finally, the small sample size may be the source of a representativeness bias. However, as the respondents came from all over France, we can assume that the collected opinions provided a broad and relevant view of the different practice contexts of the surgeons (e.g., private and public hospitals, less than or more than ten years of experience, usage or not of navigation systems or robots).

Another limitation was related to the surgery itself. Indeed, the arthroplasty procedure can be performed either as primary or revision surgery. As the number of younger patients undergoing primary knee arthroplasty increases, the risk of early prosthesis failure requiring revision is expected to increase due to their higher functional demands. Revision knee arthroplasties have poorer outcomes in terms of patient satisfaction and longevity. There is a need for improvement in primary and revision knee arthroplasty [33]. The use of 3DP CIs with individualized implementation is one possible strategy, as it can be used either at the time of the initial implantation or the time of the revision itself. In primary TKA, a CI can provide an individual fit that optimizes coverage and closely mimics the normal kinematics of the patient [34]. In revision surgery, problems that occur during the lifetime of the prosthesis and bone loss amounts could be taken into account. This information about bone quality (e.g., bone thickness, fracture risk zone during surgery, etc.) could be added to the design of the CI [35]. In our study, we did not have the opportunity to distinguish between these two cases. Primary knee arthroplasty and revision need to be addressed in a separate study.

Our study was conducted in France and, therefore, reflects the opinions of French surgeons. Identifying concerns about 3DP in other countries could be of interest to examine any agreements or divergences of opinion.

## 5. Conclusions

The objective of our study was to examine knee surgeons’ perceptions of 3DP at a time of significant expansion of this technology. Our study showed that there was no opposition to its implementation. However, approximately half of the surgeons indicated that they were waiting for validated results. They also asked questions about the entire supply chain, including hospitals, insurance companies, and manufacturers. Full adoption of 3DP will require advances in all areas of joint replacement.

## Figures and Tables

**Table 1:** Categorization of motivations with verbal examples (translated from French).

Category	Examples of Surgeons’ Motivations
Surgery	“Yes if combined with a kinematic alignment and a CT cone beam” “Not interested, this is not the right target”
Cost	“The benefit remains to be demonstrated in the light of the extra cost” “Costs ?; […] Why not though”
Logistics	“[…] Manufacturing time and availability, especially in hospitals” “Ordering issues […]”
Material	“Mechanical strength?” “Lifespan issue”
Time	“very attractive but increases the time needed for preoperative preparation” “don’t think it’s useful (time-consuming + costly)”
Regulatory	“Difficult to obtain marketing authorization?” “Issues dealing with resistance’s official approvals”
Customization	“Interest in customization” “[…] Potential customized biomechanical interest for our patients”

**Table 2:** Categorization of motivations according to the surgical phase with verbal examples (translated from French).

Category	Examples of Surgeons’ Motivations
Pre-operative	“It will deeply depend on manufacturing’s criteria”
Intra-operative	“It [3DP prosthesis] has to be compatible with operator’s habits and skills”
Post-operative	“Ok if as reliable as standard prostheses”

**Table 3:** Opinions expressed regarding surgeons’ sociodemographics and technology usage. Positive indicates both strongly and weakly positive. Negative indicates both strongly and weakly negative.

	3DP Expressed Opinion			
	Negative, *n* = 22 ^1^	Positive, *n* = 51 ^1^	Total, *n* = 73 ^1^	*p*-Value ^2^
Experience (years)				0.8
≤10	9 (41%)	19 (37%)	28 (38%)	
More than 10	13 (59%)	32 (63%)	45 (62%)	
Working structure				0.8
Private	9 (41%)	19 (37%)	28 (38%)	
Public	13 (59%)	32 (63%)	45 (62%)	
Number of prostheses per year				0.4
≤100	16 (73%)	32 (63%)	48 (66%)	
>100	6 (27%)	19 (37%)	25 (34%)	
Use of planning software				>0.9
Yes	11 (50%)	25 (49%)	36 (49%)	
No	11 (50%)	26 (51%)	37 (51%)	
Use of navigation system or robot				0.022
Yes	3 (14%)	21 (41%)	24 (33%)	
No	19 (86%)	30 (59%)	49 (67%)	
Extra surgical time				0.034
Yes	13 (59%)	42 (82%)	55 (75%)	
No	9 (41%)	9 (18%)	18 (25%)	

^1^* n* (%). ^2^ Chi-squared test of independence.

**Table 4:** Expressed motivations related to the justification of opinions. Due to the multiple motivations expressed in one response, the percentages for “Classification of Motivation” and “Motivation and Stage of Surgery” are relative to the number of surgeons who provided motivations in their primary opinions. Positive indicates both strongly and weakly positive. Negative indicates both strongly and weakly negative.

	Positive		Negative		Total
	*n*	%	*n*	%	*n*
Explicit motivation	51	70%	22	30%	73
YES	24	47%	15	68%	39
NO	27	53%	7	32%	34
Classification of motivation					
Surgery	11	46%	7	47%	18
Costs	3	13%	2	13%	5
Logistics	2	8%	3	20%	5
Materials	2	8%	5	33%	7
Customization	4	17%	-	-	-
Time	3	13%	1	7%	4
Regulatory	-	-	3	20%	-
Sub-total	25	-	21	-	46
Motivation and stage of surgery					
Pre	15	63%	9	60%	24
Intra	3	13%	1	7%	4
Post	7	29%	6	40%	13
Sub-total	25	-	16	-	41

## Data Availability

Data supporting the results are available from the corresponding author on reasonable request.

## Appendix A

**Original Text in French**	***Proposed Translation into English***
Pas intéressé, pas la cible je pense	*Not interested, this is not the right target*
Génial	*Brilliant*
Je ne vois pas l’intérêt	*Don’t see the point*
Oui si combinée à un alignement cinématique et un cône beam tdm	*Yes if combined with a kinematic alignment and a CT cone beam*
Bénéfice reste à prouver à la vue du surcout	*The benefit remains to be demonstrated in the light of the extra cost*
Le coût ?, Fiabilité ?, Mais pourquoi pas …	*Costs ?, Reliability ?, Why not though …*
Délai de fabrication et accessibilité notamment centre hospitalier	*Manufacturing time and availability, especially in hospitals*
Difficultés de commande liées à imagerie complémentaire nécessaire Tom etc…	*Ordering issues […]*
Résistance mécanique ?	*Mechanical strength ?*
Problème de la durée de vie	*Lifespan issue*
très séduisant mais allonge les délais de confection en préopératoire	*very attractive but increases the time needed for preoperative preparation*
ne pense pas que ce soit utile (chronophage + coûteux)	*don’t think it’s useful (time-consuming + costly)*
Autorisation de mise sur le marché difficile à obtenir?	*Difficult to obtain marketing authorization?*
Problème d’homologationx de résistance	*Issues dealing with resistance’s official approvals*
Intérêt de personnalisation	*Interest in customization*
[…] Probable intérêt biomécanique personnalisée pour nos patients.	*[…] Potential customized biomechanical interest for our patients*
Ça va beaucoup dépendre de s critères sur lesquels on se base pour la fabrication.	*It will deeply depend on manufacturing’s criteria*
Elle doit pouvoir “cohabiter” avec la compétence et les habitudes de l’opérateur.	*It [3DP prosthesis] has to be compatible with operator’s habits and skills*
Ok si performance identique aux prothèses classiques	*Ok if as reliable as standard prostheses*
Aucun avis	*No opinion*
Rien	*Nothing*
excellent	*excellent*
ça peut être le futur	*it may be the future*
c’est une bonne idée en raison de la rapidité de la réalisation de la prothèse	*good idea given the speed of the prosthesis manufacturing process*
inutile	*useless*
négligeable	*insignificant*
quel est le genou normal du patient	*what is the patient’s normal knee*
bonne solution si rencontre les standards actuels	*good solution if it meets current standards*
pas mal	*not bad*
fragilité	*fragility*
dubitatif quant à la qualité des matériaux	*dubious about the quality of materials*
prudence, prudence	*prudence, prudence*
